# Association of maternal longitudinal haemoglobin concentrations with adverse pregnancy outcomes: A prospective cohort study

**DOI:** 10.7189/jogh.16.04168

**Published:** 2026-05-22

**Authors:** Xueyin Wang, Xiaosong Zhang, Juan Juan, Di Gao, Huixia Yang, Meihua Zhang, Xu Chen, Xietong Wang, Yuyan Ma, Yue Teng, Haixia Meng, Xiaoqing Wang, Qiuhong Yang, Lin Xu, Shufan Shan

**Affiliations:** 1 Department of Obstetrics and Gynaecology and Reproductive Medicine, Peking University First Hospital, Beijing, China; 2 Department of Obstetrics and Gynaecology, Taiyuan Maternity and Child Health Hospital, Taiyuan, China; 3 Department of Obstetrics, Tianjin Central Obstetrics and Gynaecology Hospital, Tianjin, China; 4 Department of Obstetrics and Gynaecology, Shandong Provincial Hospital Affiliated with Shandong First Medical University, Jinan, China; 5 Department of Obstetrics and Gynaecology, Qilu Hospital of Shandong University, Jinan, China; 6 Department of Nutrition, Haidian District Maternal and Child Health Care Hospital, Beijing, China; 7 Department of Obstetrics and Gynaecology, The Affiliated Hospital of Inner Mongolia Medical University, Hohhot, China; 8 Department of Obstetrics and Gynaecology, The Third People’s Hospital of Datong, Datong, China; 9 Department of Obstetrics, Jinan Maternity and Child Care Hospital, Jinan, China; 10 Department of Obstetrics and Gynaecology, The Affiliated Hospital of Qingdao University, Qingdao, China; 11 Department of Obstetrics and Gynaecology, Affiliated Hospital of Chifeng University, Chifeng, China

## Abstract

**Background:**

Maternal haemoglobin (Hb) is a potential biomarker of maternal and neonatal health. Little is known about trimester-specific associations between maternal Hb concentrations and adverse pregnancy outcomes. Here, we assessed the magnitude and shape of these associations and further estimated developmental trajectories of maternal Hb concentrations during pregnancy.

**Methods:**

In this prospective, observational cohort study, we enrolled pregnant women at 11–13 weeks of gestation from 13 hospitals of northern China between October 2020 and June 2024. We classified maternal Hb concentrations in the first, second, and third trimester (*i.e.* 11–13, 24–27, and 36–40 weeks’ gestation, respectively) into three groups: <110 g/L, 110–129 g/L, and ≥130 g/L, respectively. We used logistic regression models, restricted cubic spline analysis, and group-based trajectory models to examine the association between maternal Hb concentrations and pregnancy outcomes.

**Results:**

Among 10 201 participants, maternal Hb concentrations of ≥130 g/L in the first, second, and third trimester were associated with increased odds of gestational diabetes mellitus and hypertension in pregnancy. Maternal Hb concentrations of <110 g/L in the second trimester were associated with higher odds of postpartum haemorrhage (odds ratio (OR) = 1.38; 95% confidence interval (CI) = 1.04–1.83). Maternal Hb concentrations of ≥130 g/L in the third trimester were associated with higher odds of small-for-gestational-age (OR = 1.48; 95% CI = 1.23–1.79) and low birth weight (OR = 1.55; 95% CI = 1.11–2.16). We further identified a U-shaped relationship between maternal Hb concentrations in the third trimester and small-for-gestational age (*P*-value for nonlinearity = 0.002). Lastly, high Hb trajectories during pregnancy were associated with higher odds of caesarean delivery (OR = 1.21; 95% CI = 1.10, 1.34), small-for-gestational age (OR = 1.41; 95% CI = 1.16, 1.72) and low birth weight (OR = 1.46; 95% CI = 1.03, 2.05).

**Conclusions:**

Both higher and lower maternal Hb concentrations were associated with higher odds of adverse pregnancy outcomes. Our findings emphasise the importance of longitudinal monitoring of maternal Hb concentrations throughout pregnancy.

**Registration:**

ClinicalTrials.gov: NCT04486456.

Anaemia during pregnancy is a prominent public health concern worldwide, particularly in low- and middle-income settings [1]. An estimated 36% of pregnant women aged 15–49 years had anaemia globally in 2019, ranging from 15% in the high-income countries to 52% in west and central Africa [2]. A recent systematic review and meta-analysis reported the prevalence of anaemia among pregnant women in China to be 30.7%, with higher estimates in rural areas and in economically less developed western region [3]. Anaemia during pregnancy contributes to several adverse pregnancy outcomes, such as higher risk of pre-eclampsia, caesarean delivery, preterm birth, low birth weight (LBW), small-for-gestational-age (SGA), low five-minute Apgar score, and neonatal morbidity and perinatal death [4,5].

Growing evidence has reported a U-shaped curve between maternal Hb concentrations and the risk of adverse pregnancy outcomes, but these associations may vary by the timing of Hb measurement during pregnancy [6–8]. A prospective study of northwest Chinese women found U-shaped associations between maternal Hb concentrations in the third trimester with the risk of LBW and SGA, but identified no significant associations between pregnancy outcomes and maternal Hb concentrations measured in the first and second trimesters [9]. A recent systematic review and meta-analysis of 148 studies concluded that low maternal Hb measured during the first trimester was correlated with elevated risk of LBW, preterm birth, SGA, and stillbirth, while low maternal Hb concentrations in the second trimester were only associated with preterm birth and stillbirth, rather than LBW and SGA [7]. Another retrospective cohort study of pregnant African-American adolescents indicated that high Hb concentrations measured in the second trimester were associated with a higher incidence of preterm birth [10], whereas a prior meta-analysis revealed that maternal Hb above 140 g/L in third trimester decreased the risk of preterm birth by 50% [11].

Studies on prospectively repeated measurements of maternal Hb concentrations in the whole pregnancy, however, remain scarce. Since maternal Hb is one of routinely measured clinical parameters during pregnancy, understanding its exact relations with pregnancy outcomes may contribute to improving maternal and neonatal health by effective strategies and interventions. Therefore, we aimed to assess the magnitude and shape of associations of maternal Hb concentrations in the first, second, and third trimesters with adverse pregnancy outcomes in northern Chinese women, and further estimate developmental trajectories of maternal Hb concentrations during pregnancy and their associations with adverse pregnancy outcomes.

## METHODS

### Study design and participants

We conducted this prospective, observational, multicentre cohort study between October 2020 and June 2024 at 13 hospitals in seven provincial-level administrative divisions of northern China, including Beijing Municipality, Tianjin Municipality, Hebei Province, Shanxi Province, Shandong Province, Shannxi Province, and Inner Mongolia Autonomous Region. We enrolled women at 11–13 weeks of gestation who received antenatal care services in the study hospitals, provided they were ≥18 years old and planned to receive routine antenatal care and be hospitalised for delivery at the study hospitals. We excluded participants with severe chronic disease before pregnancy and severe mental illnesses. We further included only participants with singleton pregnancy; measurements of maternal Hb concentrations at 11–13 weeks, 24–27 weeks, 32–35 weeks and 36–40 weeks of gestation; and with complete medical records.

We performed our sample size calculation in PASS 2021 (NCSS, LLC., Kaysville, Utah, USA). Given a rate of anaemia during pregnancy of 19.8% in Chinese hospitals [12], we required 3168 participants to achieve 90% power at a two-sided significance level of 0.05 to detect an odds ratio (OR) of 1.5 with a two-sided confidence interval (CI) width of 0.5.

### Data collection

Trained investigator conducted face-to-face interviews with each participant at enrolment using a structured questionnaire that collected demographic characteristics, lifestyle behaviours, and medical and obstetric history. Demographics included maternal age, education (junior school and below, senior high school, and college or graduate school), employment (‘employed’ or ‘unemployed’), average household income per month (RMB<3000, 3000–5000, 5000–10000, and >10000), ethnicity (Han or others), drinking during pregnancy (‘yes’ or ‘no’), smoking before pregnancy (‘yes’ or ‘no’), passive smoking during pregnancy (‘yes’ or ‘no’), parity (0, 1 and ≥2), and iron supplementation (‘yes’ or ‘no’). For medical data, the weight and height of each pregnant woman at enrolment before 13 weeks of gestation and each routine antenatal visit. We calculated participants’ pre-pregnancy BMI as the weight in kilograms divided by the square of height measured in meters, and gestational weight gain was defined as the difference between the weight measured in the last antenatal visit before delivery and the weight measured at enrolment. Participants were followed up at 24–27, 32–35, and 36–40 weeks of gestation.

### Measurement and classification of maternal Hb concentrations

Maternal Hb concentrations were extracted from medical records at 11–13 weeks, 24–27 weeks, 32–35 weeks and 36–40 weeks of gestation. According to the Chinese criteria [13], we defined anaemia during pregnancy as maternal Hb <110 g/L. Given a U-shaped association between maternal Hb concentrations and adverse pregnancy outcomes identified by previous studies [8,14], we categorised maternal Hb concentrations in the first, second, and third trimester (11–13, 24–27, and 36–40 weeks’ gestation, respectively) into three groups: <110 g/L, 110–129 g/L, and ≥130 g/L, respectively. We also extracted Hb concentrations at 16–19 weeks of gestation from medical records to further estimate developmental trajectories of maternal Hb concentrations during pregnancy. Since Hb concentrations are not included in regular antenatal care at 16–19 weeks of gestation, 3310 measurements were missing. All study hospitals are tertiary institutions whose laboratories regularly undergo quality control oversight by the National Center for Clinical Laboratories, which ensures that all tests are conducted according to uniform criteria and standards.

### Definition of adverse pregnancy outcomes

Adverse pregnancy outcomes consisted of gestational diabetes mellitus (GDM), hypertension in pregnancy (HIP), caesarean delivery, LBW, macrosomia, SGA, large-for-gestational-age (LGA), postpartum haemorrhage, and neonatal ward admission. GDM was diagnosed based on the International Association of Diabetes and Pregnancy Study Groups criteria [15], which recommend diagnosis when any one of the following values is met or exceeded during the 75 g oral glucose tolerance test at 24–28 weeks of gestation: 5.1 mmol/L for fasting, 10.0 mmol/L at one hour, and 8.5 mmol/L at two hours. HIP was defined as a clinic systolic blood pressure ≥140 mm Hg and/or a diastolic blood pressure ≥90 mm Hg [16]. LBW and macrosomia were defined as birth weight <2500 g and ≥4000 g, respectively. According to the Chinese neonatal birth weight curve, SGA and LGA was indicated by birth weight less than 10th percentile or greater than 90th percentile for a specific completed gestational age by sex, respectively [17]. Postpartum haemorrhage was defined as blood loss of >500 mL for vaginal delivery, and >1000 mL for caesarean delivery [18]. Information on pregnancy outcomes was extracted from medical records.

### Statistical analysis

We presented demographic characteristics and pregnancy outcomes as frequencies and percentages for categorical variables, or medians and interquartile ranges for continuous variables (due to the non-normal distribution of data, as checked by the Kolmogorov–Smirnov tests), comparing them across groups using χ^2^ and Mann–Whitney U tests, respectively. We established univariable and multivariable logistic regression models to estimate ORs and their 95% CIs for pregnancy outcomes across categories of maternal Hb concentrations measured at 11–13, 24–27, and 36–40 weeks of gestation, respectively. Multivariable models were adjusted for age, pre-pregnancy BMI, education, employment, average household income per month, ethnicity, drinking during pregnancy, smoking before pregnancy, passive smoking during pregnancy, parity, and iron supplementation [19]. Pregnant women with Hb concentrations of 110–129 g/L were used as the reference group.

We also used restricted cubic spline logistic regression models to assess dose-response relationship of maternal Hb concentrations measured at 11–13, 24–27, and 36–40 weeks of gestation on adverse pregnancy outcomes by treating the median level of Hb concentrations as the reference, using four knots located at the 5th, 35th, 65th, and 95th percentiles of the distribution. We also constructed group-based trajectory models (GBTMs) using five measurements of maternal Hb concentrations (11–13 weeks, 16–19 weeks, 24–27 weeks, 32–35 weeks and 36–40 weeks of gestation) to identify the developmental trajectory of maternal Hb concentrations and analysed the associations between developmental trajectory of maternal Hb concentrations and the odds of pregnancy outcomes. The optimal trajectory model was selected based on the lowest values of the Bayesian information criterion, Akaike information criterion, and log-likelihood, as well as the highest relative entropy. The GBTMs were fitted using ‘PROC TRAJ’ in SAS [20], which employs maximum likelihood estimation and is robust to missing data under the missing at random assumption. All participants with at least one Hb measurement were included, with the model using all available data. All *P*-values are two-sided, with statistical significance set at a level of 0.05. We performed all analyses in SAS, version 9.4 (SAS Institute, Cary, NC).

## RESULTS

### Participant characteristics

A total of 18 416 pregnant women were enrolled in this study. After excluding women who were twin or multiple pregnancies (n = 82), diagnosed with anaemia (n = 473), diabetes (n = 58) or hypertension before pregnancy (n = 45), diagnosed with placenta previa and/or placenta accreta spectrum disorders (n = 140), lack of medical records or any of four Hb measurements (n = 7431). The final analysis was thus restricted to 10 201 participants, among whom 2.7%, 14.5%, and 6.5% were diagnosed with anaemia at 11–13, 24–27, and 36–40 weeks of gestation, respectively, with 43.8%, 9.5%, and 25.9% having maternal Hb concentrations ≥130 g/L (Table S1 in the **Online Supplementary Document**). The median of maternal age was 31 years old, and the median of gestational age at delivery was 39 weeks. The median pre-pregnancy BMI and gestational weight gain were 22.07 kg/m^2^ and 12.00 kg, respectively. A total of 88.9% of participants had college or graduate school education; 88.2% were employed; 54.5% had average household income greater than 10 000 RMB per month; 96.4% were Han ethnicity. Most of participants did not smoke before pregnancy (98.6%), experienced passive smoking during pregnancy (93.5%), and did not drink (93.5%). A further 75.2% were nulliparous, and 32.9% had iron supplementation at enrolment (**Table 1**).

**Table 1:** Characteristics and pregnancy outcomes of study participants (n = 10 201)

Maternal characteristics	Values
Maternal age in years, MD (IQR)	31 (28–34)
Pre-pregnancy BMI in kg/m^2^, MD (IQR)	22.07 (20.13–24.46)
Gestational age at delivery, week, MD (IQR)	39 (38–40)
Gestational weight gain in kg, MD (IQR)	12.00 (9.10–15.00)
Education	
*Junior school and below*	387 (3.8)
*Senior high school*	749 (7.3)
*College or graduate school*	9065 (88.9)
Employment	
*Employed*	9000 (88.2)
*Unemployed*	1201 (11.8)
Average household income per month in RMB	
*<3000*	319 (3.1)
*3000–5000*	978 (9.6)
*5000–10000*	3348 (32.8)
*>10000*	5556 (54.5)
Ethnicity	
*Han*	9834 (96.4)
*Other*	367 (3.6)
Drinking during pregnancy	
*No*	9541 (93.5)
*Yes*	660 (6.5)
Smoking before pregnancy	
*No*	10059 (98.6)
*Yes*	142 (1.4)
Passive smoking during pregnancy	
*No*	9535 (93.5)
*Yes*	666 (6.5)
Parity	
*0*	7669 (75.2)
*1*	2318 (22.7)
*≥2*	214 (2.1)
Iron supplementation	
*No*	6848 (67.1)
*Yes*	3353 (32.9)
**Pregnancy outcomes**	
Caesarean delivery	3534 (34.6)
Small-for-gestational-age	537 (5.3)
Low birth weight	187 (1.8)
Large-for-gestational-age	621 (6.1)
Macrosomia	450 (4.4)
Gestational diabetes mellitus	2018 (19.8)
Hypertension in pregnancy	730 (7.2)
Postpartum haemorrhage	332 (3.3)
Neonatal ward admission	525 (5.1)

BMI – body mass index, MD – median, IQR – interquartile range

*Presented as n (%) unless specified otherwise.

Overall, 2018 (19.8%) and 730 (7.2%) pregnant women were diagnosed with GDM and HIP, whereas 332 (3.3%) had postpartum haemorrhage (**Table 1**). A total of 537 (5.3%), 187 (1.8%), 621 (6.1%), and 450 (4.4%) of neonates were identified as SGA, low birthweight, LGA, and macrosomia, respectively, while 525 (5.1%) were admitted to neonatal wards (Table S2 in the **Online Supplementary Document**).

### Associations between maternal Hb concentrations and pregnancy outcomes

After adjusting for potential confounders, maternal Hb concentrations ≥130 g/L at 11–13 weeks of gestation were associated with an increased odds of GDM (OR = 1.48; 95% CI = 1.34–1.64) and HIP (OR = 1.23; 95% CI = 1.06–1.44). Maternal Hb concentrations ≥130 g/L at 24–27 weeks of gestation were associated with increased odds of caesarean delivery (OR = 1.20; 95% CI = 1.03–1.39), GDM (OR = 1.22; 95% CI = 1.03–1.43), and HIP (OR = 1.59; 95% CI = 1.28–1.98), while maternal Hb concentrations <110 g/L were associated with lower odds of HIP (OR = 0.73; 95% CI = 0.57–0.94) and higher odds of postpartum haemorrhage after multiple adjustments (OR = 1.59; 95% CI = 1.28–1.98). Women with Hb concentrations ≥130 g/L at 36–40 weeks of gestation had higher odds of SGA (OR = 1.48; 95% CI = 1.23–1.79), LBW (OR = 1.55; 95% CI = 1.11–2.16), GDM (OR = 1.56; 95% CI = 1.40–1.74) and HIP (OR = 1.42; 95% CI = 1.20–1.67), whereas maternal Hb concentrations <110 g/L were associated with an increased odds of caesarean delivery (OR = 1.23; 95% CI = 1.03–1.47) (**Table 2**).

**Table 2:** Association between maternal haemoglobin concentrations and pregnancy outcomes (n = 10 201), OR (95% CI)

	First trimester (11–13 weeks)		Second trimester (24–27 weeks)		Third trimester (36–40 weeks)	
	**Model 1**	**Model 2**	**Model 1**	**Model 2**	**Model 1**	**Model 2**
**Caesarean delivery**						
Hb <110 g/L	0.82 (0.63–1.07)	0.84 (0.63–1.11)	1.00 (0.89–1.12)	0.98 (0.86–1.11)	1.25 (1.06–1.48)	1.23 (1.03–1.47)
Hb 110–129 g/L	ref	ref	ref	ref	ref	ref
Hb ≥130 g/L	1.17 (1.07–1.27)	1.10 (1.00–1.20)	1.27 (1.11–1.45)	1.20 (1.03–1.39)	1.14 (1.04–1.26)	1.11 (1.00–1.23)
**Small-for-gestational-age**						
Hb <110 g/L	1.24 (0.74–2.09)	1.13 (0.67–1.92)	1.13 (0.89–1.43)	1.00 (0.78–1.28)	1.00 (0.68–1.46)	0.93 (0.63–1.36)
Hb 110–129 g/L	ref	ref	ref	ref	ref	ref
Hb ≥130 g/L	1.30 (1.09–1.55)	1.09 (0.91–1.30)	1.09 (0.81–1.46)	1.03 (0.76–1.38)	1.67 (1.39–2.01)	1.48 (1.23–1.79)
**Low birthweight**						
Hb <110 g/L	1.14 (0.50–2.61)	1.32 (0.56–3.10)	0.80 (0.51–1.25)	0.80 (0.50–1.29)	0.83 (0.42–1.63)	0.86 (0.41–1.78)
Hb 110–129 g/L	ref	ref	ref	ref	ref	ref
Hb ≥130 g/L	0.92 (0.69–1.24)	0.89 (0.64–1.22)	1.17 (0.74–1.86)	1.27 (0.78–2.06)	1.51 (1.11–2.06)	1.55 (1.11–2.16)
**Large-for-gestational-age**						
Hb <110 g/L	0.87 (0.49–1.53)	0.77 (0.43–1.37)	1.16 (0.93–1.45)	1.06 (0.85–1.33)	1.08 (0.78–1.50)	0.96 (0.69–1.34)
Hb 110–129 g/L	ref	ref	ref	ref	ref	ref
Hb ≥130 g/L	1.36 (1.16–1.61)	1.17 (0.99–1.39)	0.93 (0.69–1.24)	0.85 (0.63–1.15)	1.03 (0.85–1.24)	0.92 (0.76–1.11)
**Macrosomia**						
Hb <110 g/L	0.65 (0.32–1.34)	0.71 (0.34–1.47)	0.97 (0.74–1.27)	0.99 (0.75–1.31)	0.79 (0.52–1.21)	0.78 (0.50–1.20)
Hb 110–129 g/L	ref	ref	ref	ref	ref	ref
Hb ≥130 g/L	1.08 (0.89–1.31)	1.04 (0.85–1.27)	0.92 (0.66–1.29)	0.84 (0.59–1.19)	0.95 (0.76–1.19)	0.91 (0.73–1.15)
**Gestational diabetes mellitus**						
Hb <110 g/L	0.94 (0.68–1.30)	0.96 (0.69–1.34)	0.98 (0.85–1.13)	0.97 (0.84–1.12)	1.11 (0.91–1.36)	1.15 (0.94–1.41)
Hb 110–129 g/L	ref	ref	ref	ref	ref	ref
Hb ≥130 g/L	1.51 (1.36–1.66)	1.48 (1.34–1.64)	1.20 (1.02–1.41)	1.22 (1.03–1.43)	1.59 (1.42–1.77)	1.56 (1.40–1.74)
**Hypertension in pregnancy**						
Hb <110 g/L	0.81 (0.47–1.37)	0.84 (0.49–1.43)	0.68 (0.53–0.88)	0.73 (0.57–0.94)	0.84 (0.59–1.18)	0.85 (0.60–1.21)
Hb 110–129 g/L	ref	ref	ref	ref	ref	ref
Hb ≥130 g/L	1.24 (1.06–1.44)	1.23 (1.06–1.44)	1.67 (1.34–2.07)	1.59 (1.28–1.98)	1.40 (1.19–1.65)	1.42 (1.20–1.67)
**Postpartum haemorrhage**						
Hb <110 g/L	1.26 (0.66–2.41)	1.10 (0.53–2.27)	1.56 (1.18–2.05)	1.38 (1.04–1.83)	1.13 (0.74–1.74)	1.07 (0.68–1.68)
Hb 110–129 g/L	ref	ref	ref	ref	ref	ref
Hb ≥130 g/L	1.31 (1.05–1.64)	1.10 (0.87–1.38)	1.07 (0.73–1.56)	0.97 (0.65–1.44)	1.03 (0.80–1.32)	0.90 (0.70–1.17)
**Neonatal ward admission**						
Hb <110 g/L	0.78 (0.41–1.48)	0.66 (0.32–1.35)	0.98 (0.76–1.27)	0.86 (0.66–1.13)	0.94 (0.64–1.37)	0.81 (0.55–1.21)
Hb 110–129 g/L	ref	ref	ref	ref	ref	ref
Hb ≥130 g/L	1.33 (1.12–1.59)	1.11 (0.93–1.34)	1.17 (0.88–1.55)	1.07 (0.79–1.44)	1.17 (0.96–1.42)	1.00 (0.82–1.23)

CI – confidence interval, Hb – haemoglobin, OR – odds ratio, ref – reference

*Model 1 was the unadjusted model. Model 2 was adjusted for age, pre-pregnancy body mass index, education, employment, average household income per month, ethnicity, drinking during pregnancy, smoking before pregnancy, passive smoking during pregnancy, parity, and iron supplementation.

### Dose-response relationship of maternal Hb concentrations and pregnancy outcomes

**Figure 1:**
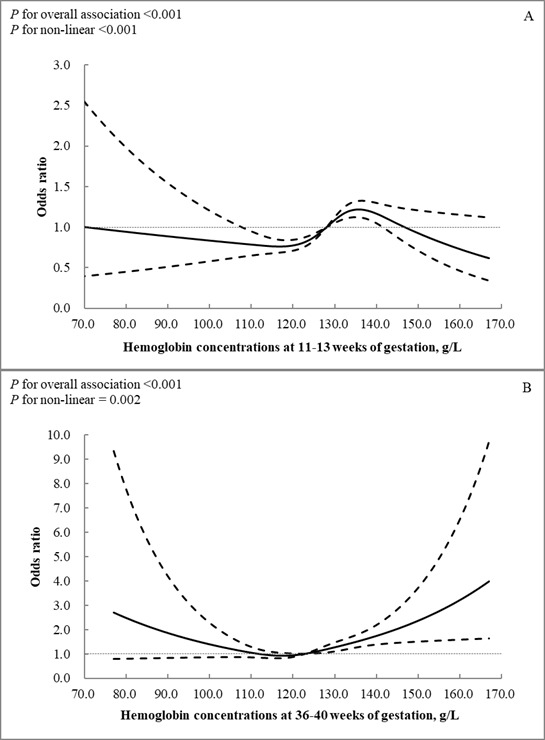
ORs and 95% CIs for maternal hemoglobin concentrations with gestational diabetes mellitus (**Panel A**) and small-for-gestational-age (**Panel B**). Adjusted for age, pre-pregnancy body mass index, education, employment, average household income per month, ethnicity, drinking during pregnancy, smoking before pregnancy, passive smoking during pregnancy, parity, and iron supplementation.

The restricted cubic spline analysis showed a significant nonlinear association of maternal Hb concentrations at 11–13 weeks of gestation with GDM after adjustment for potential confounders (*P*-value for overall association <0.001, *P*-value for nonlinearity <0.001) (**Figure 1**, Panel A). We noted a U-shaped association of maternal Hb concentrations at 36–40 weeks of gestation with the odds of SGA after adjustment for potential confounders (*P*-value for overall association <0.001, *P*-value for nonlinearity = 0.002), suggesting that both lower and higher levels of Hb concentrations were associated with elevated odds of SGA compared with the median of maternal Hb concentrations of 123 g/L (**Figure 1**, Panel B). We found no other dose-response relationship between maternal Hb concentrations and other pregnancy outcomes (data not shown).

### Associations between maternal Hb concentration trajectories and pregnancy outcomes

**Figure 2:**
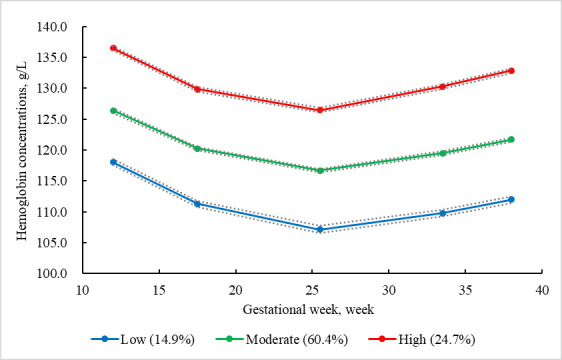
Trajectories of maternal haemoglobin concentrations.

The GBTM identified three maternal Hb trajectories (**Figure 2**): low (n = 1523, 14.9%), moderate (n = 6156, 60.4%), and high (n = 2522, 24.7%). The model demonstrated excellent classification accuracy and good fit (Table S3 in the **Online Supplementary Document**), with average posterior probabilities of 0.860, 0.890, and 0.897 for the low, moderate, and high trajectory group, respectively, and with respective odds of correct classification of 34.9, 5.3, and 26.6. After controlling for potential confounders, the high Hb concentration group had a greater risk of caesarean delivery (OR = 1.21; 95% CI = 1.10–1.34), SGA (OR = 1.41; 95% CI = 1.16–1.72) and LBW (OR = 1.46; 95% CI = 1.03–2.05) than the moderate Hb concentration group (**Table 3**).

**Table 3:** Association between maternal haemoglobin concentrations and pregnancy outcomes analysed by grouped-based trajectory models (n = 10 201), OR (95% CI)*

	Low	Moderate	High
**Caesarean delivery**			
Model 1	0.99 (0.88–1.11)	ref	1.21 (1.09–1.33)
Model 2	0.98 (0.87–1.10)	ref	1.21 (1.10–1.34)
**Small-for-gestational-age**			
Model 1	1.09 (0.84–1.41)	ref	1.58 (1.30–1.91)
Model 2	1.02 (0.78–1.33)	ref	1.41 (1.16–1.72)
**Low birthweight**			
Model 1	0.66 (0.40–1.10)	ref	1.35 (0.98–1.86)
Model 2	0.73 (0.43–1.24)	ref	1.46 (1.03–2.05)
**Large-for-gestational-age**			
Model 1	1.08 (0.86–1.36)	ref	1.03 (0.85–1.25)
Model 2	1.01 (0.80–1.27)	ref	0.93 (0.76–1.13)
**Macrosomia**			
Model 1	0.93 (0.70–1.23)	ref	1.01 (0.81–1.26)
Model 2	0.90 (0.67–1.21)	ref	0.93 (0.73–1.17)
**Postpartum haemorrhage**			
Model 1	1.08 (0.80–1.47)	ref	0.92 (0.70–1.20)
Model 2	1.04 (0.76–1.43)	ref	0.80 (0.61–1.05)
**Neonatal ward admission**			
Model 1	0.81 (0.61–1.07)	ref	1.26 (1.03–1.53)
Model 2	0.74 (0.55–1.00)	ref	1.10 (0.89–1.35)

CI – confidence interval, OR – odds ratio, ref – reference

*Model 1 was the unadjusted model. Model 2 was adjusted for age, pre-pregnancy body mass index, education, employment, average household income per month, ethnicity, drinking during pregnancy, smoking before pregnancy, passive smoking during pregnancy, parity, and iron supplementation.

## DISCUSSION

This prospective cohort study of Chinese pregnant women found that maternal Hb concentrations ≥130 g/L at 36–40 weeks of gestation were associated with higher risk of SGA and LBW, and further identified a U-shaped relationship between maternal Hb concentrations and the risk of SGA. Maternal Hb concentrations ≥130 g/L at 11–13, 24–27, and 36–40 weeks of gestation were associated with an increased risk of GDM and HIP. We also observed the significant association of maternal Hb concentrations ≥130 g/L at 24–27 and <110 g/L at 36–40 weeks of gestation with an increased risk of caesarean delivery, while maternal Hb concentrations <110 g/L at 24–27 weeks of gestation were associated with greater odds of postpartum haemorrhage.

Our finding of a U-shaped relationship between maternal Hb concentrations at 36–40 weeks of gestation and SGA risk suggests that both lower and higher Hb concentrations are associated with an increased risk of SGA. A prior meta-analysis of 11 studies also reported a U-shaped curve between maternal Hb and SGA [21]. Another prospective study conducted in northwest China showed that maternal Hb levels in the third trimester were associated with SGA in an extended U-shaped pattern [9]. Gustavo *et al*. [22] utilised the first available Hb measurement during pregnancy, and indicated that women with Hb of 110–130 g/L had the minimal risk of SGA. We also found that maternal Hb concentrations ≥130 g/L at 36–40 weeks of gestation were associated with higher risk of LBW, which aligns with previous studies indicating that high Hb levels were related to lower neonatal birth weight and an increased risk of LBW [23,24].

We observed a significant association between high maternal Hb concentrations and an increased risk of SGA and LBW only in the third, and not first and second trimesters, which is in line with the results of a prospective study conducted among women in central China [25]. By contrast, a previous retrospective cohort analysis of US pregnant women found that women with higher Hb levels during the first and second, rather than the third trimester, had an increased risk of SGA [26]. These discrepancies may be partly attributed to differences in study design, ethnicity, and sample sizes across studies. The potential mechanisms underlying the relationship between maternal Hb and the risk of SGA and LBW might be partly attributed to poor plasma volume expansion and increased blood viscosity. Plasma volume expansion is a critical physiological alteration that could promote utero-placental circulation and thus facilitate foetal growth [27]. However, high Hb concentrations during pregnancy, especially in the third trimester, may reflect insufficient plasma volume expansion which could cause increased blood viscosity and further reduce placental blood ﬂow velocity as well as impede the delivery of nutrients and oxygen, thereby limiting foetal growth [6,8,28].

Another important finding of this study is that maternal Hb concentrations ≥130 g/L in the first, second, and third trimesters were associated with an increased risk of GDM and HIP. In agreement with these results, a previous meta-analysis also reported that high Hb could increase the risk of GDM by 52% in the first trimester [29]. Another prospective UK cohort study also indicated the higher Hb levels at both early and late pregnancy increased the odds of GDM by 51% and 35%, respectively [30]. Similarly, a prospective observational study of non-diabetic Chinese women suggested that pregnant women in the highest Hb quartile had a significantly higher incidence of GDM compared with those in the lowest Hb quartile [31]. As for HIP, previous evidence also indicates a significant association between high Hb concentrations in the first and second trimester and an increased risk of HIP among nulliparous women [32,33], which aligns with our results. Elevated iron status and Hb levels have been proposed to be implicated in glycaemic status during pregnancy and pathophysiology of GDM and HIP [34]. Besides blood viscosity and placenta perfusion, excessive iron concentrations may produce reactive oxygen and induce oxidative stress *via* the Fenton reaction, leading to dysfunction in *β*-cells and impaired insulin secretion and increased insulin resistance [35]. Furthermore, a rapid increase of iron and oxygen could lead to oxidative stress, and excess membrane lipid peroxidation and ferroptosis at the maternal-foetal interface, which results in DNA damage of placental cells, shallow endovascular invasion of extravillous cytotrophoblast, and suboptimal remodelling of the maternal spiral arteries [30,36,37].

We also found that maternal Hb concentrations <110g/L in the second trimester were associated with 38% greater odds of postpartum haemorrhage. In line with our results, a systematic review and meta-analysis of 95 studies showed that maternal Hb ≤110 g/L at any time during pregnancy was associated with elevated odds of postpartum haemorrhage [38]. Data from China’s Hospital Quality Monitoring System also indicated that all anaemia categories during pregnancy, including mild, moderate, or severe anaemia, were associated with an increased risk of postpartum haemorrhage compared with no anaemia [39]. A prospective cohort study conducted in Pakistan, Nigeria, Tanzania, and Zambia showed that prebirth anaemia was strongly correlated with higher risk of postpartum haemorrhage [40]. The possible mechanisms linking anaemia in pregnancy and postpartum haemorrhage might be explained by that decreased myometrial contractility, impaired coagulation caused by damaged transport of Hb and oxygen, and tissue enzyme and cellular dysfunction could together induce uterine atony [41].

Our findings further indicate that both maternal Hb concentrations ≥130 g/L at 24–28 and <110 g/L at 36–40 weeks of gestation are associated with greater odds of caesarean delivery. A recent cross-sectional study from western China also exhibited a U-shaped relationship between maternal Hb concentrations at first trimester and caesarean delivery, which partially supported our findings [42]. However, evidence regarding the trimester-specific association between Hb concentrations and caesarean delivery is limited. Here, we note that, despite the robust temporal design, causality cannot be inferred from the associations reported in this observational study. The modest effect sizes observed for caesarean delivery suggest that Hb concentration represents one of contributing factors, and alternative mechanisms, including haemoconcentration secondary to inadequate plasma volume expansion, warrant further investigation.

This study has important implications for public health and clinical practice, as these findings indicated that both higher and lower maternal Hb concentrations were associated with elevated odds of adverse pregnancy outcomes, which may contribute to early identification and prevention. This highlights the necessity for longitudinal monitoring of maternal Hb concentrations throughout pregnancy to identify women at high risk of adverse pregnancy outcomes. The main strength of this study is the prospectively repeated measurements of maternal Hb concentrations across the continuum of pregnancy, which allowed us to evaluate trimester-specific associations between maternal Hb concentrations and adverse pregnancy outcomes, as well as examine maternal Hb trajectories during the entire pregnancy. Additional strengths include a relatively large sample size, adjustments for multiple potential confounders, and the use of several statistical models, including restricted cubic spline analysis and GBTMs, for exploring the association between maternal Hb concentrations and pregnancy outcomes.

Several factors should be considered when generalising our findings. Our participants were recruited from urban medical centres and were predominantly of Han ethnicity, employed, residents of urban areas, and had higher levels of education. Therefore, our findings are most directly applicable to analogous populations. Second, we did not have access to information about iron status, iron markers of inflammation and nutrition intake during pregnancy. As these factors are known to influence Hb concentrations, it is possible that Hb is merely a proxy for these underlying conditions, which could confound the associations observed in our analyses. Finally, the assessment of multiple adverse pregnancy outcomes in relation to maternal Hb concentrations may increase the risk of false-positive findings due to multiple comparisons, so our results should be interpreted with caution. Future large-scale studies are required to address these gaps and fully elucidate the role of maternal Hb levels in pregnancy outcomes.

## CONCLUSIONS

Our findings indicate that maternal Hb concentrations ≥130 g/L in the third trimester were associated with higher risk of SGA and LBW, with a U-shaped relationship between maternal Hb concentrations and SGA. We also found that maternal Hb concentrations ≥130 g/L in the first, second, and third trimester were associated with an increased risk of GDM and HIP. Our findings emphasise the importance of longitudinal monitoring of maternal Hb concentrations throughout pregnancy, as both high and low levels are associated with adverse pregnancy outcomes.

## Additional material


Online Supplementary Document
