# Supplementary material for: Association of maternal longitudinal haemoglobin concentrations with adverse pregnancy outcomes: A prospective cohort study
Source: J Glob Health. 2026 May 22;16:04168. doi: 10.7189/jogh.16.04168 (PMC13196492; doi:10.7189/jogh.16.04168)
Supplement: Online Supplementary Document [file jogh-16-04168-s001.pdf]

Supplement to: Wang X, Zhang X, Juan J, Gao D, Yang H, Zhang M, Chen X, Wang X, Ma Y, Teng Y, Meng H, Wang X, Yang Q, Xu L, Shan S. Association of maternal longitudinal hemoglobin concentrations with adverse pregnancy outcomes: A prospective cohort study. J Glob Health. 2026;1:04168.

Table S1. Maternal hemoglobin categories in the first, second and third trimesters (n=10,201)

Maternal hemoglobin concentrations	First trimester (11-13 weeks)	Second trimester (24-27 weeks)	Third trimester (36-40 weeks)
Hb <110 g/L	280 (2.7)	1479 (14.5)	664 (6.5)
Hb 110-129 g/L	5451 (53.4)	7753 (76.0)	6895 (67.6)
Hb ≥130 g/L	4470 (43.8)	969 (9.5)	2642 (25.9)

Table S2. Proportion of pregnancy outcomes by maternal hemoglobin categories (n=10,201), %

	Hb <110 g/L		Hb 110-129 g/L		Hb ≥130 g/L		*P* value
	n	Proportion (95% CI)	n	Proportion (95% CI)	n	Proportion (95% CI)	
Gestational diabetes mellitus							
First trimester (11-13 weeks)	45	16.1 (11.8, 20.4)	923	16.9 (15.9, 17.9)	1050	23.5 (22.3, 24.7)	<0.001
Second trimester (24-27 weeks)	284	19.2 (17.2, 21.2)	1515	19.5 (18.7, 20.4)	219	22.6 (20.0, 25.2)	0.167
Third trimester (36-40 weeks)	128	19.3 (16.3, 22.3)	1219	17.7 (16.8, 18.6)	671	25.4 (23.7, 27.1)	<0.001
Hypertension in pregnancy							
First trimester (11-13 weeks)	15	5.4 (2.7, 8.0)	358	6.6 (5.9, 7.2)	357	8.0 (7.2, 8.8)	0.001
Second trimester (24-27 weeks)	73	4.9 (3.8, 6.0)	548	7.1 (6.5, 7.6)	109	11.3 (9.3, 13.2)	<0.001
Third trimester (36-40 weeks)	37	5.6 (3.8, 7.3)	455	6.6 (6.0, 7.2)	238	9.0 (7.9, 10.1)	<0.001
Cesarean delivery							
First trimester (11-13 weeks)	81	28.9 (23.6, 34.2)	1812	33.2 (32.0, 34.5)	1641	36.7 (35.3, 38.1)	<0.001
Second trimester (24-27 weeks)	504	34.1(31.7, 36.5)	2646	34.1 (33.1, 35.2)	384	39.6 (36.6, 42.7)	0.002
Third trimester (36-40 weeks)	257	38.7 (35.0, 42.4)	2311	33.5 (32.4, 34.6)	966	36.6 (34.7, 38.4)	0.007
Small-for-gestational-age							
First trimester (11-13 weeks)	16	1.4 (3.0, 8.4)	254	4.7 (4.1, 5.2)	267	6.0 (5.3, 6.7)	0.124
Second trimester (24-27 weeks)	85	5.8 (4.6, 6.9)	398	5.1 (4.6, 5.6)	54	5.6 (4.1, 7.0)	0.488
Third trimester (36-40 weeks)	30	4.5 (2.9, 6.1)	313	4.5 (4.1, 5.0)	194	7.4 (6.4, 8.3)	0.017
Low birthweight							
First trimester (11-13 weeks)	6	2.1 (0.5, 3.8)	103	1.9 (1.5, 2.3)	78	1.7 (1.4, 2.1)	0.740
Second trimester (24-27 weeks)	22	1.5 (0.9, 2.1)	144	1.0 (1.6, 2.2)	21	2.2 (1.3, 3.1)	0.539
Third trimester (36-40 weeks)	9	1.4 (0.5, 2.2)	113	1.6 (1.3, 1.9)	65	2.5 (1.9, 3.1)	0.071
Large-for-gestational-age							
First trimester (11-13 weeks)	13	4.6 (2.2, 7.1)	290	5.3 (4.7, 5.9)	318	7.1 (6.4, 7.9)	0.412
Second trimester (24-27 weeks)	102	6.9 (5.6, 8.2)	465	6.0 (5.5, 6.5)	54	5.6 (4.1, 7.0)	0.881
Third trimester (36-40 weeks)	43	6.5 (4.6, 8.4)	415	6.0 (5.5, 6.6)	163	6.2 (5.3, 7.1)	0.974
Macrosomia							
First trimester (11-13 weeks)	8	2.9 (0.9, 4.8)	235	4.3 (3.8, 4.9)	207	4.6 (4.0, 5.3)	0.233
Second trimester (24-27 weeks)	64	4.3 (3.3, 5.4)	346	4.5 (4.0, 4.9)	40	4.1 (2.9, 5.4)	0.972

Third trimester (36-40 weeks)	24	3.6 (2.2, 5.0)	312	4.5 (4.0, 5.0)	114	4.3 (3.5, 5.1)	0.562
Postpartum hemorrhage							
First trimester (11-13 weeks)	10	3.6 (1.4, 5.8)	156	2.9 (2.4, 3.3)	166	3.7 (3.2, 4.3)	0.231
Second trimester (24-27 weeks)	68	4.6 (3.5, 5.7)	233	3.0 (2.6, 3.4)	31	3.2 (2.1, 4.3)	0.006
Third trimester (36-40 weeks)	24	3.6 (2.2, 5.0)	221	3.2 (2.8, 3.6)	87	3.3 (2.6, 4.0)	0.789
Neonatal ward admission							
First trimester (11-13 weeks)	10	3.6 (1.4, 5.8)	248	4.6 (4.0, 5.1)	267	6.0 (5.3, 6.7)	0.002
Second trimester (24-27 weeks)	74	5.0 (3.9, 6.1)	394	5.1 (4.6, 5.6)	57	5.9 (4.4, 7.4)	0.655
Third trimester (36-40 weeks)	31	4.7 (3.1, 6.3)	342	5.0 (4.5, 5.5)	152	5.8 (4.9, 6.6)	0.317

Table S3. Model fit and classification diagnostics for the trajectory model

	Overall	Group 1	Group 2	Group 3
Group size, n (%)	10,201 (100%)	1,523 (14.9%)	6,156 (60.4%)	2,522 (24.7%)
BIC	-167,577.9	-	-	-
AIC	-167,525.2	-	-	-
Log likelihood	-167,513.2	-	-	-
AvePP	-	0.860	0.890	0.897
OCC	-	34.9	5.3	26.6
Relative entropy	0.76	-	-	-

Abbreviations: BIC, Bayesian Information Criterion; AIC, Akaike Information Criterion; AvePP, Average posterior probability; OCC, Odds of correct classification.
